# Efficacy of probiotics in the prevention of VAP in critically ill ICU patients: an updated systematic review and meta-analysis of randomized control trials

**DOI:** 10.1186/s40560-020-00487-8

**Published:** 2020-10-15

**Authors:** Priyam Batra, Kapil Dev Soni, Purva Mathur

**Affiliations:** 1 Department of Microbiology, AIIMS, New Delhi, 110029 India; 2 Department of Critical and Intensive Care, JPNA Trauma Center, AIIMS, Room No. 323, New Delhi, 110029 India; 3 Department of Laboratory Medicine, JPNA Trauma Center, AIIMS, New Delhi, 110029 India

**Keywords:** Ventilator-associated pneumonia, Critical care, Probiotics, Placebo, Meta-analysis

## Abstract

**Introduction:**

Ventilator-associated pneumonia (VAP) is reported as the second most common nosocomial infection among critically ill patients with the incidence ranging from 2 to 16 episodes per 1000 ventilator days. The use of probiotics has been shown to have a promising effect in many RCTs. Our systematic review and meta-analysis were thus planned to determine the effect of probiotic use in critically ill ventilated adult patients on the incidence of VAP, length of hospital stay, length of ICU stay, duration of mechanical ventilation, the incidence of diarrhea, and the incidence of oropharyngeal colonization and in-hospital mortality.

**Methodology:**

Systematic search of various databases (such as Embase, Cochrane, and Pubmed), published journals, clinical trials, and abstracts of the various major conferences were made to obtain the RCTs which compare probiotics with placebo for VAP prevention. The results were expressed as risk ratios or mean differences. Data synthesis was done using statistical software - Review Manager (RevMan) Version 5.4 (The Cochrane Collaboration, 2020).

**Results:**

Nine studies met our inclusion criterion and were included in the meta-analysis. The incidence of VAP (risk ratio: 0.70, CI 0.56, 0.88; *P* = 0.002; *I*^2^ = 37%), duration of mechanical ventilation (mean difference −3.75, CI −6.93, −0.58; *P* 0.02; *I*^2^ = 96%), length of ICU stay (mean difference −4.20, CI −6.73, −1.66; *P* = 0.001; *I*^2^ = 84%) and in-hospital mortality (OR 0.73, CI 0.54, 0.98; *P* = 0.04; *I*^2^ = 0%) in the probiotic group was significantly lower than that in the control group. Probiotic administration was not associated with a statistically significant reduction in length of hospital stay (MD −1.94, CI −7.17, 3.28; *P* = 0.47; *I*^2^ = 88%), incidence of oro-pharyngeal colonization (OR 0.59, CI 0.33, 1.04; *P* = 0.07; *I*^2^ = 69%), and incidence of diarrhea (OR 0.59, CI 0.34, 1.03; *P* = 0.06; *I*^2^ = 38%).

**Discussion:**

Our meta-analysis shows that probiotic administration has a promising role in lowering the incidence of VAP, the duration of mechanical ventilation, length of ICU stay, and in-hospital mortality.

## Background

Ventilator-associated pneumonia (VAP) is reported as the second most common nosocomial infection among critically ill patients [1] with the incidence ranging from 2 to 16 episodes per 1000 ventilator days [2]. VAP is associated with an increase in the duration of hospitalization by 7 days, an increase in the healthcare cost by approximately 40,000 USD [3] and is reported to be the leading cause of death among nosocomial infections [4]. The pathogenesis of VAP is very complex but primarily involves bacterial translocation and colonization of the aerodigestive tract with pathogenic bacteria. This is followed by aspiration of these pathogenic micro-organisms into the lower respiratory tract thus causing pneumonia [5].

Numerous trials and studies are done to determine the best pharmacological preventive strategies inhibiting the colonization of the micro-organisms such as the use of antibiotics for selective digestive decontamination (SDD) or selective oral decontamination (SOD) or the use of probiotics. The use of antibiotics for SDD or SOD has been associated with an increase in antibiotic resistance and cost [6] but the use of probiotics as a preventive measure has been shown to have promising results in various studies [7]. Probiotics are live non-pathogenic microbes that reduce bacterial translocation by activating mucosal immunity and regulating the release of proinflammatory cytokines [5]. They also inhibit the growth of pathogenic micro-organisms by many mechanisms which include the production of various substances (such as organic acid, hydrogen peroxide, and bacteriocins), competition for nutrients, inhibition of pathogen attachment, and inhibition of the action of microbial toxins. Probiotics also stimulate the proliferation of the normal epithelium which helps maintain the mucosal defense barrier [8]. Prebiotics are non-digestible sugars that selectively stimulate the growth of certain bacterial colonies while a combination of probiotics and prebiotics is called synbiotics [9].

Seeing the promising nature of probiotics in critically ill patients, many randomized controlled trials (RCTs) have been conducted in recent years to evaluate the effectiveness of probiotics in the prevention of VAP. A recent meta-analysis performed by Su et al. in 2020 [1] and Cheng et al. in 2018 [5] showed that probiotics are efficient in decreasing the incidence of VAP. However, the meta-analysis by Su et al. included few studies [10, 11] in which the use of probiotics was not compared with placebo as a control. Also, the trials included in the study [12, 13] were of low quality. A large meta-analysis with trial sequential analysis published by Weng et al. [14] in 2017 also supported the role of probiotics in the prevention of VAP but the study included children in the patient population.

Thus, the current study was planned to determine the effect of the use of probiotics in critically ill ventilated adult patients on the incidence of VAP, length of hospital stay, length of ICU stay, duration of mechanical ventilation, the incidence of diarrhea, and the incidence of oropharyngeal colonization and in-hospital mortality.

## Methodology

### Protocol preparation and registration

A protocol for the study was prepared and has been registered in Prospero [15].

### Eligibility criteria

The protocol was prepared to include RCTs done on critically ill patients on a ventilator. RCTs selected were those which used probiotics/synbiotics in the patients of the intervention arm and used placebo in the control arm patients. Studies using any other medication besides the placebo in the control arm were not included in the study as it would have led to bias in the study.

### Data sources

Two reviewers independently made a systematic search of EMBASE, MEDLINE (Pubmed), Web of Science, and the Cochrane Central Registry of Controlled Trials (CENTRAL) from inception to February 2020, to include clinical trials conducted in humans regarding probiotics and VAP. The search was limited to studies published in English. Search terms included “critically ill” “sepsis” “trauma” “ventilation- associated” “probiotics” “synbiotics”. Abstracts of major conferences and trials database were also searched for. Bibliographies of all relevant trials, systematic reviews, and meta-analysis were also hand-scanned.

### Study selection

Two reviewers (PB and KDS) independently screened studies for inclusion depending on the eligibility criterion. Randomized control trials reporting the use of probiotics for the prevention of ventilator-associated pneumonia (VAP) in critically ill patients admitted in intensive care units (ICUs) were included in the meta-analysis. Studies reporting different types of probiotics (*Lactobacillus* spp., *Pediococcus* spp., *Leuconostoc* spp., *Bifidobacterium* spp., *Bacillus subtilis*, *Streptococcus* spp., *Ergyphilus* spp., *Bifidus* spp., *Saccharomyces* spp., *Enterococcus* spp.) alone or in combination with prebiotics were included in the study. Studies including pediatric patients or studies using probiotics as therapeutic agents or studies comparing large versus low doses of probiotics or studies comparing different types of probiotics were excluded from our study.

### Data extraction

Both authors (PB and KDS) screened and evaluated titles and available abstracts of identified citations in duplicate to determine eligibility. Full-text publication of all articles that were judged as potentially eligible by the review team was downloaded and eligibility criteria were applied to the full text of all potentially eligible trials. Any disagreement between the reviewers was resolved by consensus and any discrepancy remaining was further resolved through discussion with the arbitrator third author (PM). The Phi or kappa statistics were applied to measure the interobserver agreement regarding the eligibility of the RCTs.

Standardized form from the Cochrane Data Collection template was adapted and used to create a study-specific data abstraction form. Two reviewers (PB and KDS) extracted the data, independently and in duplicate, from all eligible studies.

### Data items

Data abstracted included demographic information, methodology, intervention details, and outcome data. The primary outcome to be studied was the incidence of VAP. Secondary outcomes that were studied included duration of mechanical ventilation, length of hospital or ICU stay (as reported), oropharyngeal colonization, the incidence of diarrhea, and mortality rate (ICU/in-hospital mortality) as reported in the study.

### Risk of bias assessment

Risk of bias was assessed by reviewers using a modified plausible quality assessment scale as recommended by the Cochrane Collaboration. This instrument included response options of “low,” “high,” or “unclear” risk of bias.

The key domains that were evaluated included random sequence generation; allocation concealment; blinding of participants/healthcare professionals/data collectors/outcome assessors/data analysts; incomplete outcome data; and reviewer’s bias. Reviewers resolved disagreement by discussion and the arbitrator adjudicated any unresolved disagreements.

### Summary measures

The incidence of ventilator-associated pneumonia was measured using risk ratio; the incidence of oro-pharyngeal colonization, the incidence of diarrhea, and in-hospital mortality were measured using odds ratio; and duration of mechanical ventilation, length of ICU stay, and length of hospital stay were measured using differences in means.

### Strategy for data synthesis

Random effect meta-analyses were used to compare similar interventions with high heterogeneity. Random effect meta-analyses included both within and between-study differences. If heterogeneity was lower, then the fixed effect meta-analyses were applied. Heterogeneity of treatment effect was assessed using Cochrane’s *Q* statistic and *I* squared statistic. Excess heterogeneity was explained using multiple approaches such as subgroup effect or sensitivity analyses. Dichotomous outcomes were reported using relative risk ratio (RR) or odds (OR) ratio whereas continuous endpoints reported in trials were calculated as weighted mean difference (MD) and standard deviation (SD). The inverse of variance was used to provide individual weightage to the studies.

### Subgroups analysis

Following subgroup analysis was performed for the primary outcome that is the incidence of VAP to explain the heterogeneity found in the studies.

Subgroup analysis of the trial grouping based on the risk of bias was done, i.e., high-risk trials vs low-risk trials.

Analysis of trials for the primary outcome for reporting in specific populations such as trauma, medical, or surgical patients.

Subgroup analysis of trials reporting micro-organisms with the trials not reporting microorganisms specifically for causation of VAP.

### Sensitivity analysis

In few studies, the duration of mechanical ventilation [16, 17], length of ICU stay [16–18], and length of hospital stay [17] were given as median (IQR) which was converted to mean ± SD for inclusion in the meta-analysis to maintain uniformity of the study results. Sensitivity analysis was done by the removal of these studies to see the change in the incidence of VAP upon removal of the concerned studies.

## Results

### Study identification and selection

**Fig. 1:**
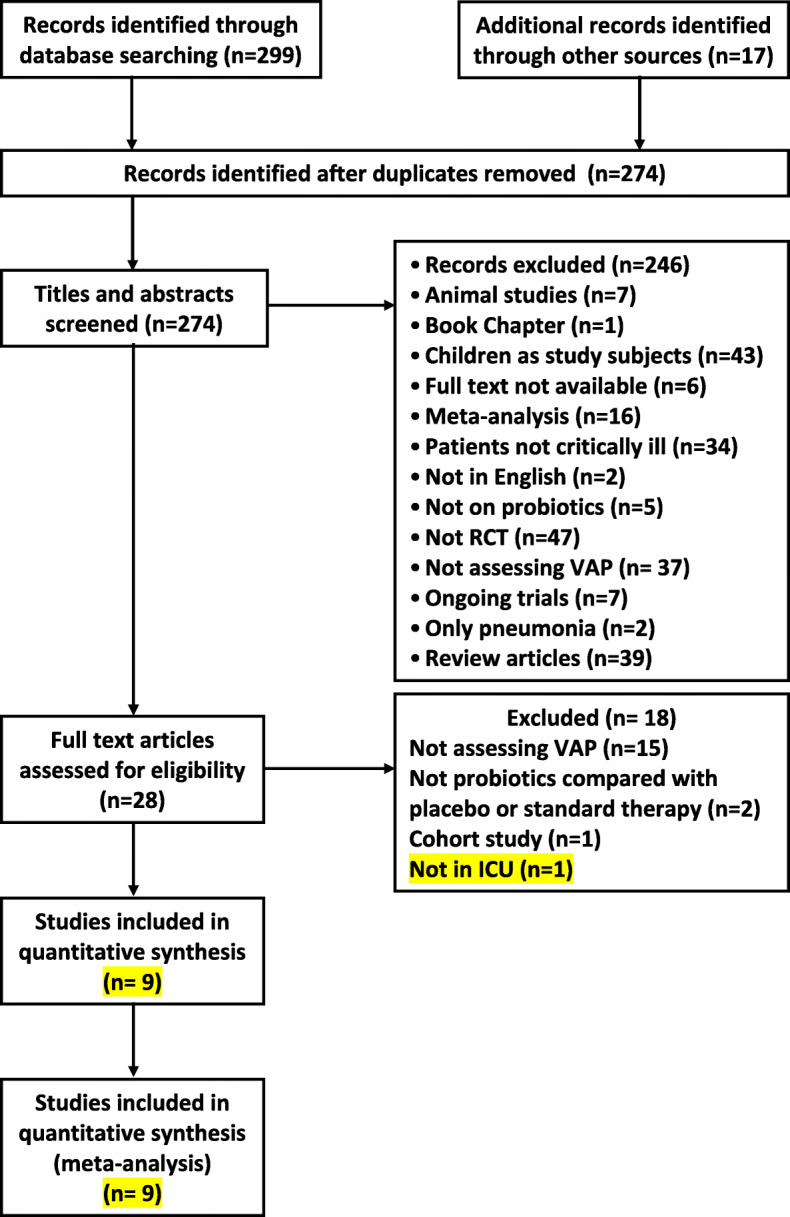
PRISMA flowchart

A systematic search of the database was made using the keywords which gave a total of 299 articles. By manual search of the references of systematic reviews and meta-analysis, 17 additional records were found. After removal of the duplicate articles, titles and abstracts of 274 publications were searched. Of these, 246 records could be easily excluded as they did not meet our inclusion criteria being animal studies (*n* = 7) or children studies (*n* = 43) or being done on patients who were not critically ill (*n* = 34). The reasons for exclusion are enlisted in Fig. 1. Full text of the remaining 28 articles was obtained and only 9 of these were found eligible for quantitative synthesis in our meta-analysis. The remaining 19 articles could be excluded as one of these was a cohort study; in 15, the outcome assessed was not VAP, in one, it was found that the study was conducted in medical wards, and in 2 of these placebos was not used in the control group. Instead, antibiotic decontamination or chlorhexidine mouth wash was being used. There was 96% agreement (Cohen’s *k* 0.92) between the two authors (PB and KDS).

### Characteristics of the included study

The characteristics of the nine studies included in the meta-analysis are given in Table 1. The table gives a detailed description of all the included studies in terms of the study design, duration of follow-up, patient population under investigation and its characteristics, intervention done, control group, outcome measured, and the definition of VAP used in the study. Most of the included studies were published in the past 10 years and their mean sample size was 125 (ranging from 52 to 259). Of the nine studies included, all [7, 16–23] the studies reported VAP in the included patients; 8 studies described length of ICU stay [7, 16–19, 21–23] and in-hospital mortality [7, 16–22]; 5 studies described duration of mechanical ventilation [7, 16, 17, 21, 22]; only 4 studies described the length of hospital stay [7, 17, 19, 22] and incidence of diarrhea [7, 19, 21, 22] and incidence of oro-pharyngeal colonization [7, 16, 17, 22]. The heterogeneity of the studies included was assessed using the Cochrane *Q* statistics.

**Table 1:** Characteristics of the study

Study	Design/duration	Participants	Intervention	Outcome	Definition of VAP
Barraud et al. [19]	Double-blind/until weaning	Adults intubated on MV > 2 days *n* = 167 SOFA score in probiotics: 9 ± 4.6 and control: 9.7 ± 4.8	Probiotic: enterally administered once a day pro-biotics Ergyphilus capsule (multispecies *Lactobacillus rhamnosus* GG, *L. casei*, *L. acidophilus,* and *Bifidobacterium bifidum*) 2 × 10^10^ CFU/d Control: Placebo Started soon after admission, continued during entire period of mechanical ventilation but not more than 28 days	Primary endpoint: 28-day mortality Secondary endpoints: 90-day mortality, the reversal of organ failure, the occurrence of ICU-acquired infections, colonization by day 28, and ICU length of stay	1. CXR + [1 sign: (1) PTS, (2) Temp ≥ 38.3 °C, (3) WBC ≥ 10,000/mm^3^] 2. Positive quantitative cultures from BAL
Giamarellos-Bourboulis et al. [20]	Double-blind/28 days	Multiorgan injuries; tracheal intubation; MV *n* = 72 APACHE II, GCS score in probiotic group: 19.36, 7.64, and control group: 19.36, 7.80	Probiotic: Synbiotic 2000 Forte*; 10^11^CFU/d by NGT/gastrostomy for 15 days Control: Placebo Started on admission to the ICU	Analyzed the microbiological and laboratory findings of patients	All of the following: (1) CXR, (2) PTS, (3) CPIS > 6
Knight et al. [17]	Double blind/28 days	Critically ill patients on ventilator; MV > 48 h *n* = 300 APACHE II score in probiotic: 17 (12-23) and control: 17 (12-22)	Probiotic: Synbiotic 2000 Forte; 10^10^CFU/d twice daily by NGT/OGT for 28 days/death/discharge Control: Placebo Started within 24 h of admission to ICU	Primary outcome: incidence of VAP Secondary outcome variables: oropharyngeal flora, ventilator days, and VAP rates per 1000 ventilator days, ICU length of stay, ICU mortality, and hospital mortality	CXR + 2 sign: (1) T ≥ 38.0 °C, (2) WBC ≥ 12,000/mm3 or ≤ 4000/mm3, (3) PTS
Kotzampassi et al. [21]	Double blind, 15 days	Severe multiple organ failure; adults; MV ≥ 48 h; life expectancy > 15 days *n* = 77 APACHE II, GCS score in probiotic group: 19.36, 7.64, and control group: 19.36, 7.80	Probiotic: Synbiotic 2000 Forte^*^; 10^11^CFU/d by NGT/gastrostomy Control: Placebo (powdered glucose polymer) Started at time of ICU admission given for 15 days	Primary endpoints: systemic infection rate during ICU stay, or the development of SIRS and MODS Secondary endpoints: Mortality, length of stay in the ICU, and number of days under mechanical ventilation	All of the following: (1) CXR, (2) PTS, (3) T ≥ 38.5 °C, (4) WBC > 12,000/mm3 or < 4000/mm3, (5) positive quantitative cultures from BAL
Mahmoodpoor et al. [22]	Double blind, 2 weeks	Critically ill adults, in ICU, MV > 48 h APACHE II score in probiotic: 24.1 ± 6.2; control: 22.8 ± 4.7	Probiotic: 1 capsule in 12 h, 10^10^ bacteria × 14 days (*Lactobacillus* species (*casei*, *acidophilus*, *rhamnosus*, *bulgaricus*), *Bifidobacterium* species (*breve*, *longum*), *Streptococcus thermophilus* administered using feeding tube; not with gavage formula) Control: Placebo (sterile maize starch powder)	Primary outcome: VAP occurrence Secondary outcomes: ICU and hospital length of stay, duration of mechanical ventilation, and complications during the study	CXR + 2 sign: (1) T ≥ 38.0 °C or ≤ 36.0 °C, (2) leukocytosis or leucopenia, (3) purulent sputum underwent BAL
Morrow et al. [7]	Double blind, not stated	Adults requiring MV > 72 h *n* = 146 APACHE II score in probiotic group: 22.7 ± 7.5, control: 23.7 ± 8	Probiotic: *L. rhamnosus* GG 2 × 10^9^ CFU/d twice daily; NGT or OGT Control: placebo Started within 24 of admission until extubated/tracheostomy placement/death	Primary outcome: Microbiologically confirmed VAP Secondary outcome: mortality; time to occurrence of VAP; durations of MV, ICU stay, and hospital stay; Clostridium difficile–associated diarrhea; other ICU-associated diarrhea; antibiotic consumption (total, VAP-specific, and C. difficile-specific); and hospital charges	CXR + 2 sign: (1) T ≥ 38.5 °C or ≤ 35.0 °C, (2) WBC ≥ 10,000/mm3 or ≤ 3000/mm3, (3) PTS
Shimizu et al. [18]	Single blind/4 week	Adults; diagnosed sepsis; on MV APACHE II score in probiotic: 19 (14-24) and control: 20 (14-26)	Probiotic: Yakult BL Seichoyaku (contains 6 × 10^8^ CFU of *B. breve* and *L. casei* with galactooligosaccharides as prebiotic) NGT daily control: Placebo doses Started within 3 days of admission	Primary outcome: infectious complications such as enteritis, ventilator-associated pneumonia (VAP), and bacteremia Secondary outcomes: mortality, fecal bacterial counts, and organic acid concentration	Pneumonia after 48-72 h of MV
Tan et al. [23]	Single blind/28 day	Closed head injury, adult, patients with severe TBI and Glasgow Coma Scale scores between 5 and 8 *n* = 52	Probiotic group: Golden Bifid containing 0.5 × 10^8^ *Bifidobacterium longum*, 0.5 × 10^7^ *Lactobacillus bulgaricus* and 0.5 × 10^7^ *Streptococcus thermophilus*. Started within 48 h of ICU admission for 21 days	VAP rate, duration of ICU stay, duration of antibiotics use, and 28-day mortality rate	CXR + 2 sign: (1) T > 38.0 °C or < 35.5 °C, (2) WBC > 12,000/mm3 or < 4000/mm3, (3) PTS, (4) positive semiquantitative cultures of TBS
Zeng et al. [16]	Open label/14 day	Critically ill adults with MV > 48 h *n* = 250 APACHE II score in probiotic: 14.7 ± 3.9; control: 16.6 ± 3.3	Probiotic group: Probiotic capsule (Medilac-S**) 0.5 g (1.5 × 10^10^) three times/day by NGT Control group: Placebo Started within 24 h of admission to the ICU given for 14 days	Primary endpoints: incidence of microbiologically confirmed VAP, proportions of eradication of colonization and acquired colonization with PPMOs in the oropharynx and stomach Secondary endpoints: duration of MV, duration of ICU stay, duration of hospital stay, mortality (in ICU, in-hospital) and number of days of antibiotic use for VAP	CXR + 2 sign: (1) T > 38.0 °C or < 35.5 °C, (2) WBC > 12,000/mm3 or < 3000/mm3 (3) TBS

*MV* mechanical ventilation, *CXR* chest X-ray, *BAL* broncho alveolar lavage, *PTS* purulent tracheal secretion, *CPIS* clinical pulmonary infection score, *WBC* white blood cells, *CFU* colony forming units, *T* temperature, *ICU* intensive care unit, *VAP* ventilator-associated pneumonia, *NGT* nasogastric tube, *OGT* orogastric tube, *TBS* tracheobronchial secretions

*Synbiotic 2000Forte contains 10^11^ CFU of *P.* pentoseceus 5–33:3, *L. mesenteroides* 32–77:1, *L. paracasei* ssp. 19, and *L. plantarum* 2362 along with inulin, betaglucan, pectin, and resistant starch as prebiotic

**Medilac S contains *Bacillus subtilis* and *Enterococcus faecalis*

Probiotics administered including the dosage and routes of administration varied in the studies. In one of the studies, a single probiotic (*Lactobacillus rhamnosus* [7]) was used, 5 studies used multiple probiotics [16, 18, 19, 22, 23], and in 3 studies a symbiotic formula (Synbiotic 2000 Forte) [17, 20, 21] was used. The severity of illness of the patients included in the study is also provided in the table.

There was variability in the definition of VAP among all studies as shown in Table 1. In two studies [7, 16] both clinical and microbiological definition was mentioned while in one study [18] no explicit definition of VAP was given. The outcome data extracted from the RCTs included in the meta-analysis are presented in Table 1.

### Risk of bias assessment

**Fig. 2:**
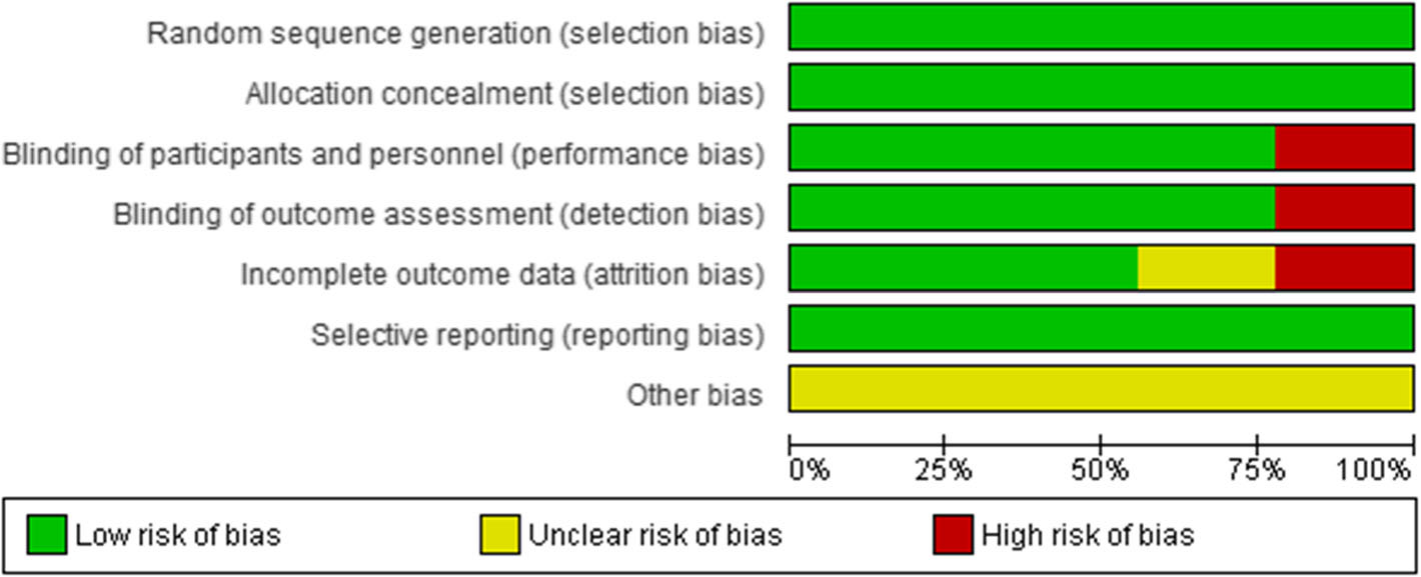
Judgements about each risk of bias item presented as percentages across all included studies

**Fig. 3:**
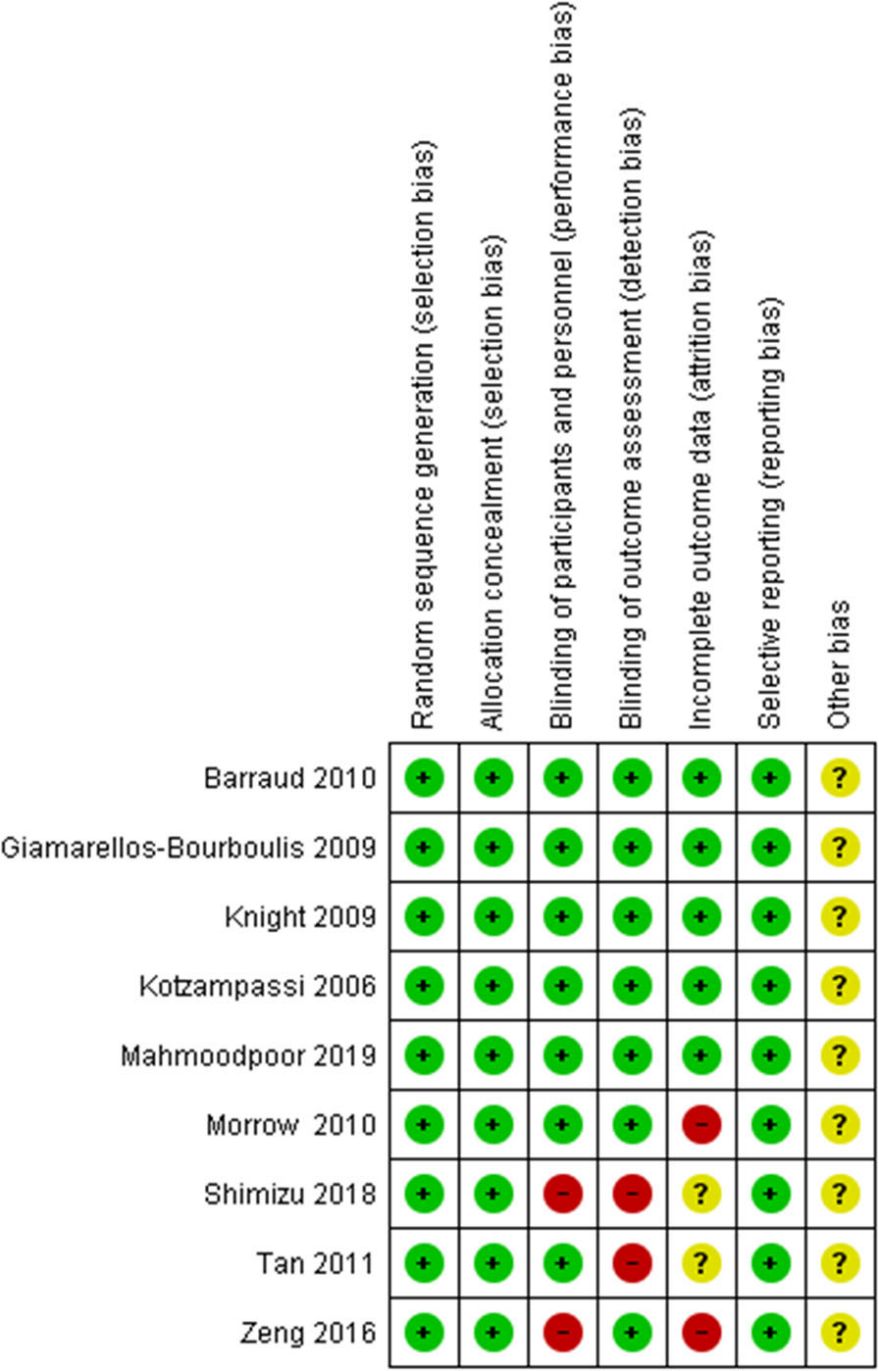
Risk of bias summary: review authors’ judgments about each risk of bias item for each included study

The risk of bias in the included studies is shown in Figs. 2 and 3. All the nine studies had a low risk of random sequence generation selection bias, allocation concealment selection bias, and selective reporting bias. Two studies had a high risk of performance bias and detection bias as they did not have good blinding of participants and personnel. The risk of selection and reporting bias was low in all the studies. However, the risk of outcome assessment detection bias was high in most of the studies. The presence of detection bias can either underestimate or overestimate the size of the effect.

### Primary outcome: incidence of VAP

**Fig. 4:**
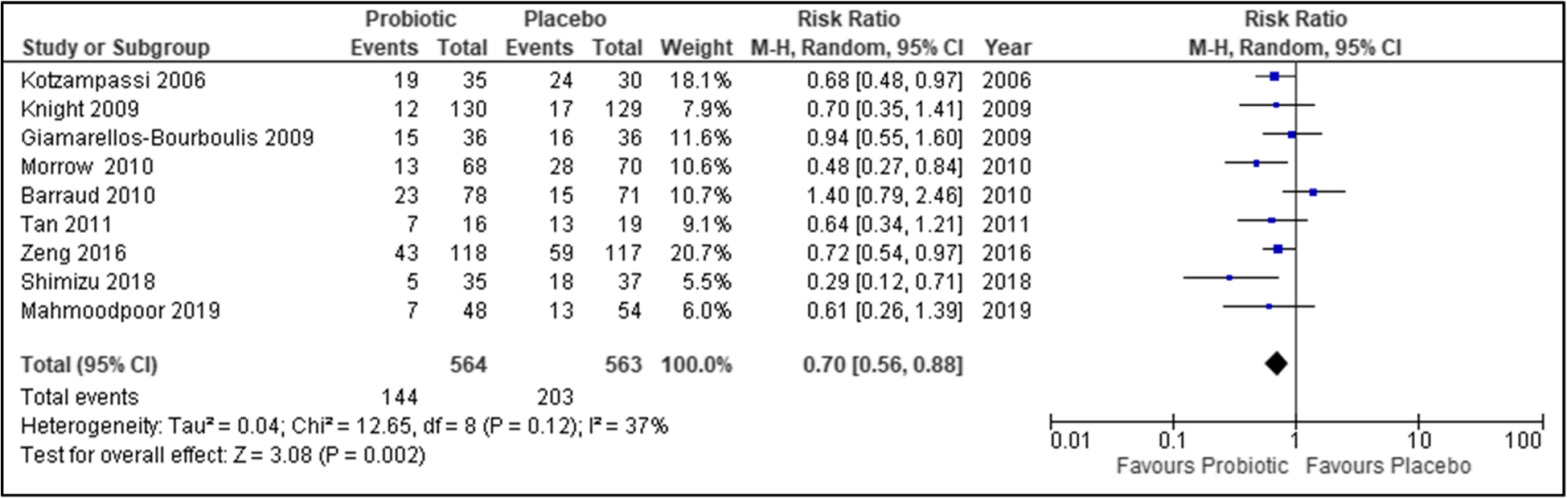
A forest plot of incidence of ventilator-associated pneumonia (VAP)

All the nine RCTs included in the study with a total patient load of 1127 (564 in probiotics group and 563 in the placebo group) reported VAP incidence as can be seen in Fig. 4. The analysis showed that the incidence of VAP in the probiotic group was significantly lower than the incidence in the control group (OR 0.70, CI 0.56, 0.88; *P* = 0.002; *I*^2^ = 37%). Low to moderate heterogeneity was seen between studies.

### Secondary outcome

The other outcomes measured duration of mechanical ventilation, length of ICU stay, length of hospital stay, the incidence of oropharyngeal colonization, the incidence of diarrhea, and in-hospital mortality. The duration of mechanical ventilation, length of ICU stay and length of hospital stay were reported either in mean ± SD or median (IQR). For comparison, all the results were taken in mean ± SD. The conversion of the median (IQR) to mean ± SD was done using the following formula [24].

Mean = (*a* + *b* + 2*m*)/4; where *a* is the low range; *b* is the high range; *m* is the median

Variance (*S*^2^) = 1/12 {[(*a*−2*m* + *b*)^2^/4] + (*b*−*a*)^2^}

#### Duration of mechanical ventilation

**Fig. 5:**
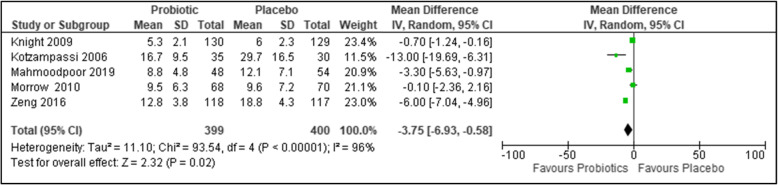
A forest plot of duration of mechanical ventilation

Five of the nine studies with a total patient size of 799 patients (399 in probiotics and 400 in the placebo arm) provided the duration of mechanical ventilation (Fig. 5). A high heterogeneity (MD −3.75, CI −6.93, −0.58; *P* = 0.02; *I*^2^ = 96%) was seen between the studies. There was a statistically significant reduction in the duration of mechanical ventilation in the probiotic group. In two studies [16, 17], the duration of mechanical ventilation was expressed in the median (IQR) and was converted into mean ± SD. If we remove both these studies for sensitivity analysis, the mean difference becomes statistically non-significant = −4.32 (−9.12, 0.49, *P* = 0.08).

#### Length of ICU stay

**Fig. 6:**
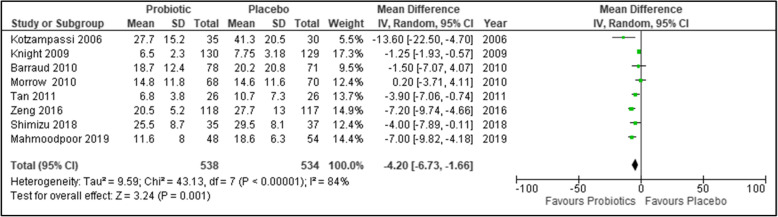
A forest plot of length of ICU stay

Eight of the studies reported length of ICU stay in 1072 patients (538 in the probiotic arm and 534 in the placebo arm) as seen in Fig. 6. A high heterogeneity (MD −4.20, CI −6.73, −1.66; *P* = 0.001; *I*^2^ = 84%) was seen between the studies. In three studies [16–18], the length of ICU stay was expressed in median (IQR) and was converted into mean ± SD. If we remove these three studies for sensitivity analysis, the mean difference still remains statistically significant −4.37 (−7.89, −0.85; *P* = 0.01).

#### Length of hospital stay

**Fig. 7:**
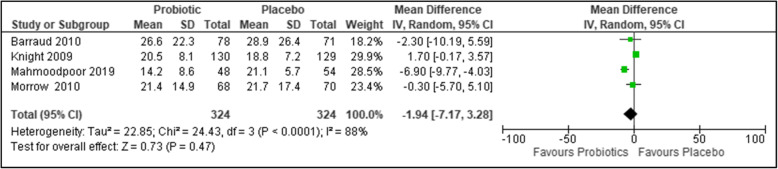
A forest plot of length of hospital stay

Four of the studies reported length of hospital stay in 648 patients (324 in the probiotic arm and 324 in the placebo arm) as seen in Fig. 7. A high heterogeneity (MD −1.94, CI −7.17, 3.28; *P* = 0.47; *I*^2^ = 88%) was seen between the studies. In one study [17], the length of hospital stay was expressed in median (IQR) and was converted into mean ± SD. If we remove this study, the mean difference remains non-significant = −3.79 (−8.47, 0.89; *P* = 0.11).

#### Incidence of oropharyngeal colonization

**Fig. 8:**
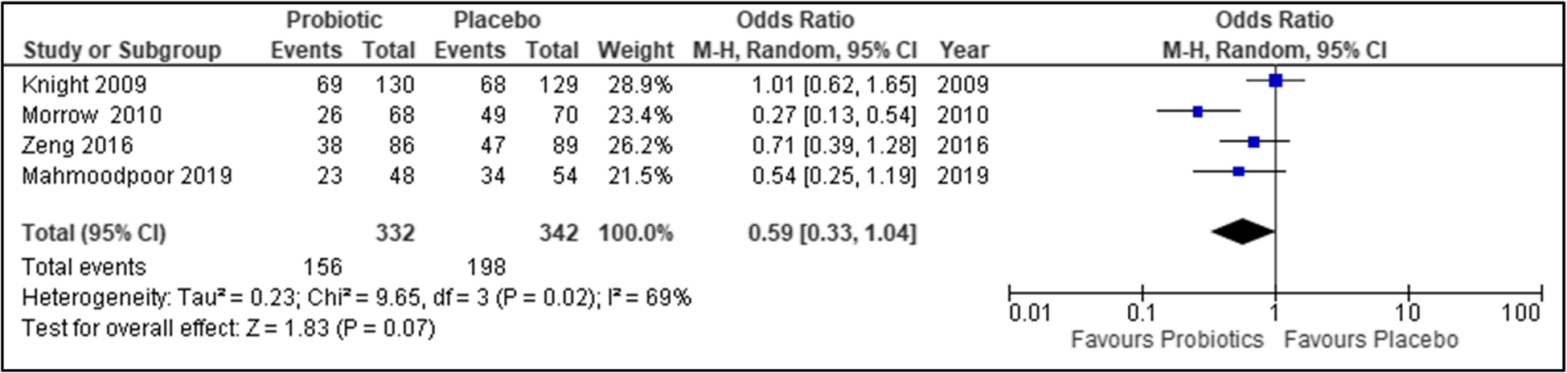
A forest plot of incidence of oro-pharyngeal colonization

Four of the studies reported oropharyngeal colonization in 674 patients (332 in the probiotic arm and 342 in the placebo arm) as seen in Fig. 8. A high heterogeneity (OR 0.59, CI 0.33, 1.04; *P* = 0.07; *I*^2^ = 69%) was seen between the studies.

#### Incidence of diarrhea

**Fig. 9:**
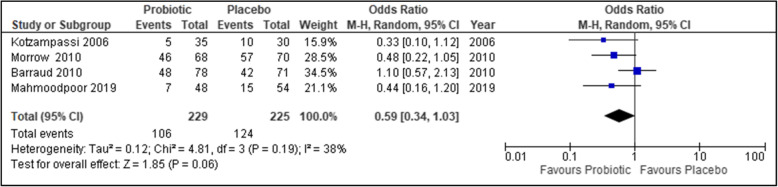
A forest plot of the incidence of diarrhea

Four of the studies reported diarrhea in 454 patients (229 in the probiotic arm and 225 in the placebo arm) as seen in Fig. 9. A moderate heterogeneity (OR 0.59, CI 0.34, 1.03; *P* = 0.06; *I*^2^ = 38%) was seen between the studies.

#### In-hospital mortality

**Fig. 10:**
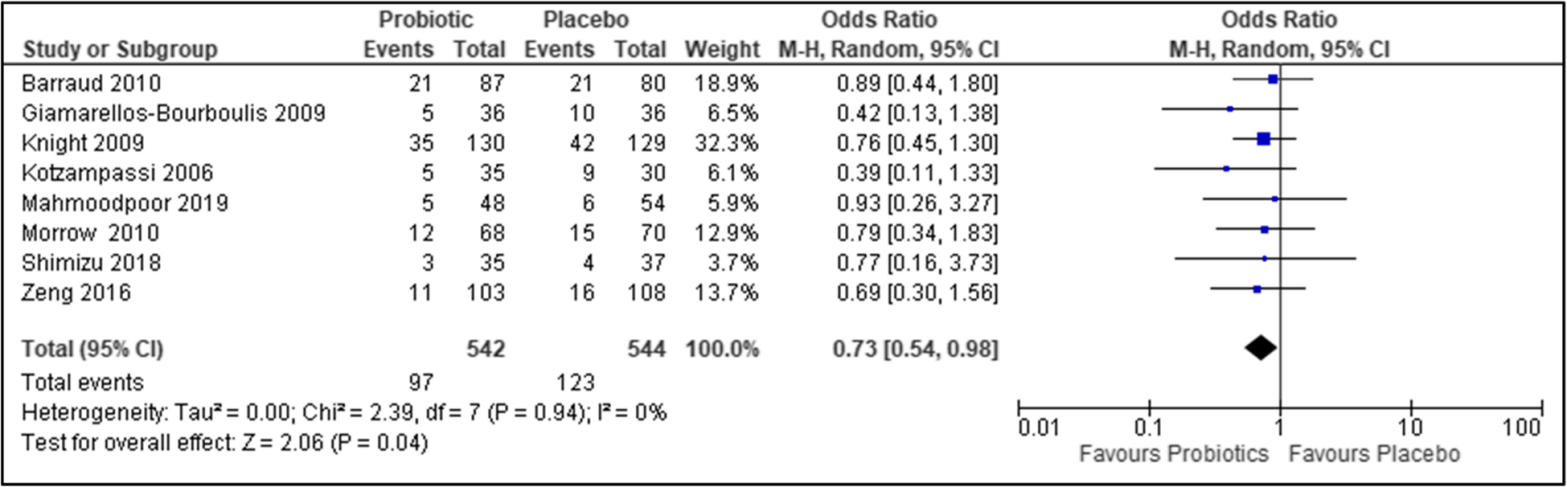
A forest plot of in-hospital mortality

Eight of the studies reported in-hospital mortality in 1086 patients (542 in the probiotic arm and 544 in the placebo arm) as shown in Fig. 10. No heterogeneity (OR 0.73, CI 0.54, 0.98; *P* = 0.04; *I*^2^ = 0%) was seen between the studies and a statistically significant difference was seen.

## Subgroup analysis

### High vs low risk of bias trials

**Fig. 11:**
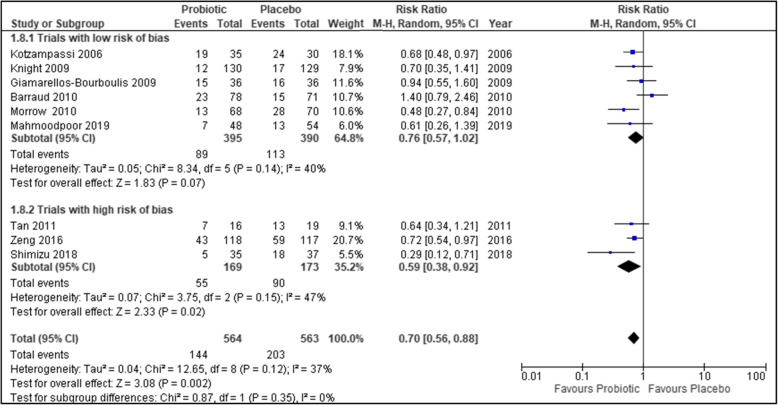
Forest plot of subgroup analysis (high vs the low risk of bias trials)

The incidence of VAP was statistically significant in trials reporting high risk of bias (RR 0.59, CI 0.38, 0.92; *P* = 0.02; *I*^2^ = 47%) while it was not significant in those reporting low risk of bias (RR 0.76, CI 0.57, 1.02; *P* = 0.07; *I*^2^ = 40%). However, the overall test for subgroup differences was not found to be statistically significant (*P* = 0.35), as can be seen in Fig. 11.

### Mixed population vs trauma population trials

**Fig. 12:**
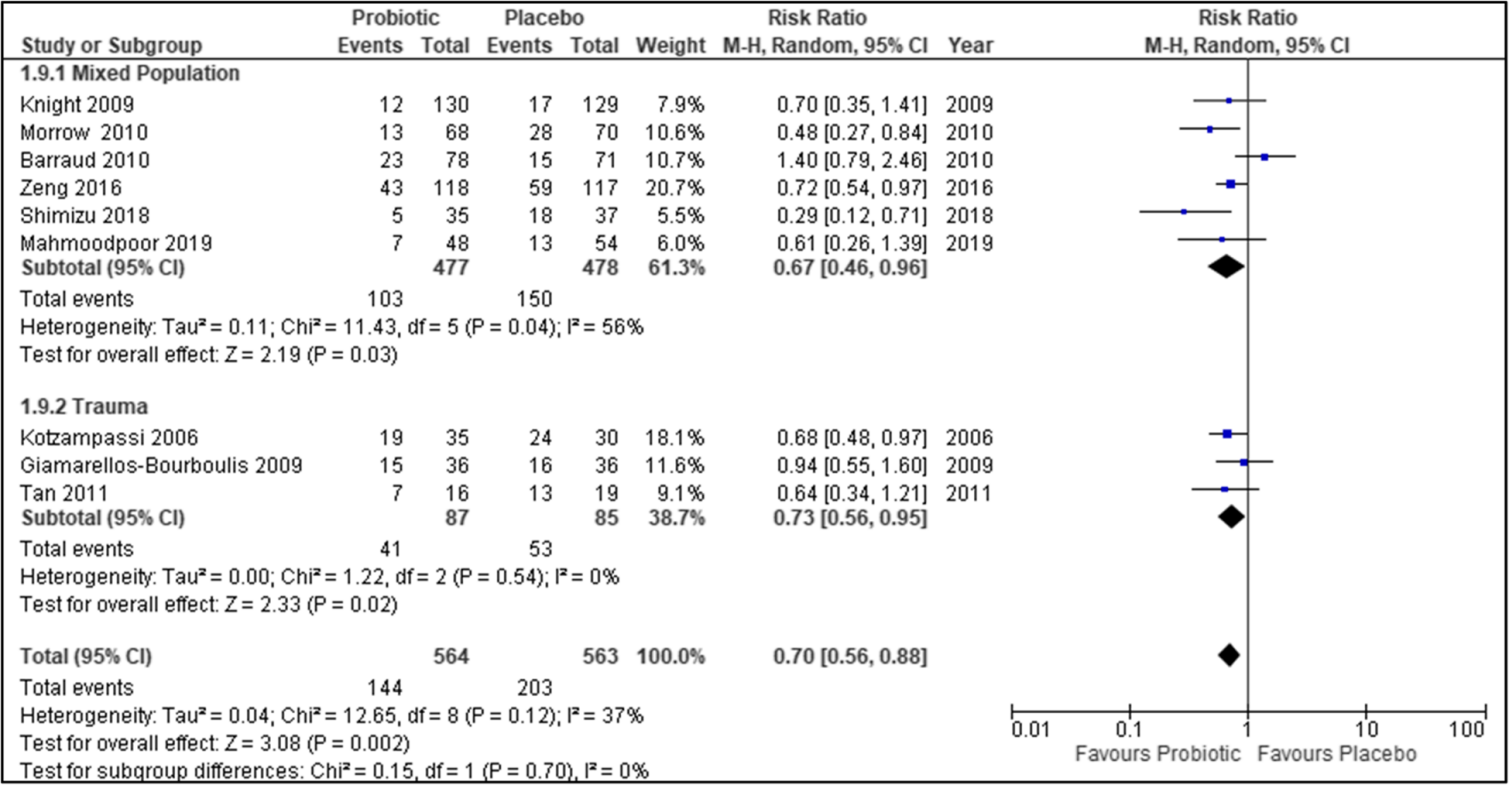
Forest plot of subgroup analysis (mixed population vs trauma population)

The incidence of VAP was similar in trials done in a mixed population of patients (RR 0.67, CI 0.46, 0.96; *P* = 0.03; *I*^2^ = 56%) as well as those done in the trauma population (RR 0.73, CI 0.56, 0.95; *P* = 0.02; *I*^2^ = 0%). The difference between the subgroups was not statistically significant (*P* = 0.70) as can be seen in Fig. 12.

### Trials reporting micro-organisms vs not reporting micro-organisms

**Fig. 13:**
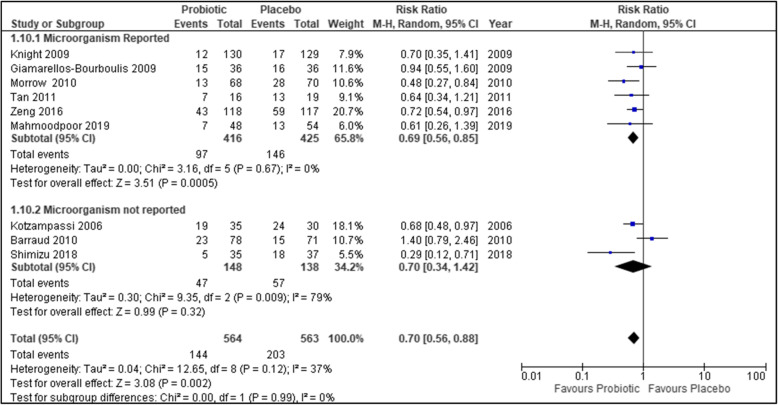
Forest plot of subgroup analysis (microorganisms vs no microorganisms reported)

The incidence of VAP was similar in trials reporting microorganisms (RR 0.69, CI 0.56, 0.85; *P* = 0.0005; *I*^2^ = 0%) as well as those not reporting microorganisms (RR 0.70, CI 0.34, 1.42; *P* = 0.32; *I*^2^ = 79%). The difference between the subgroups was not statistically significant (*P* = 0.99) as can be seen in Fig. 13.

### Publication bias

**Fig. 14:**
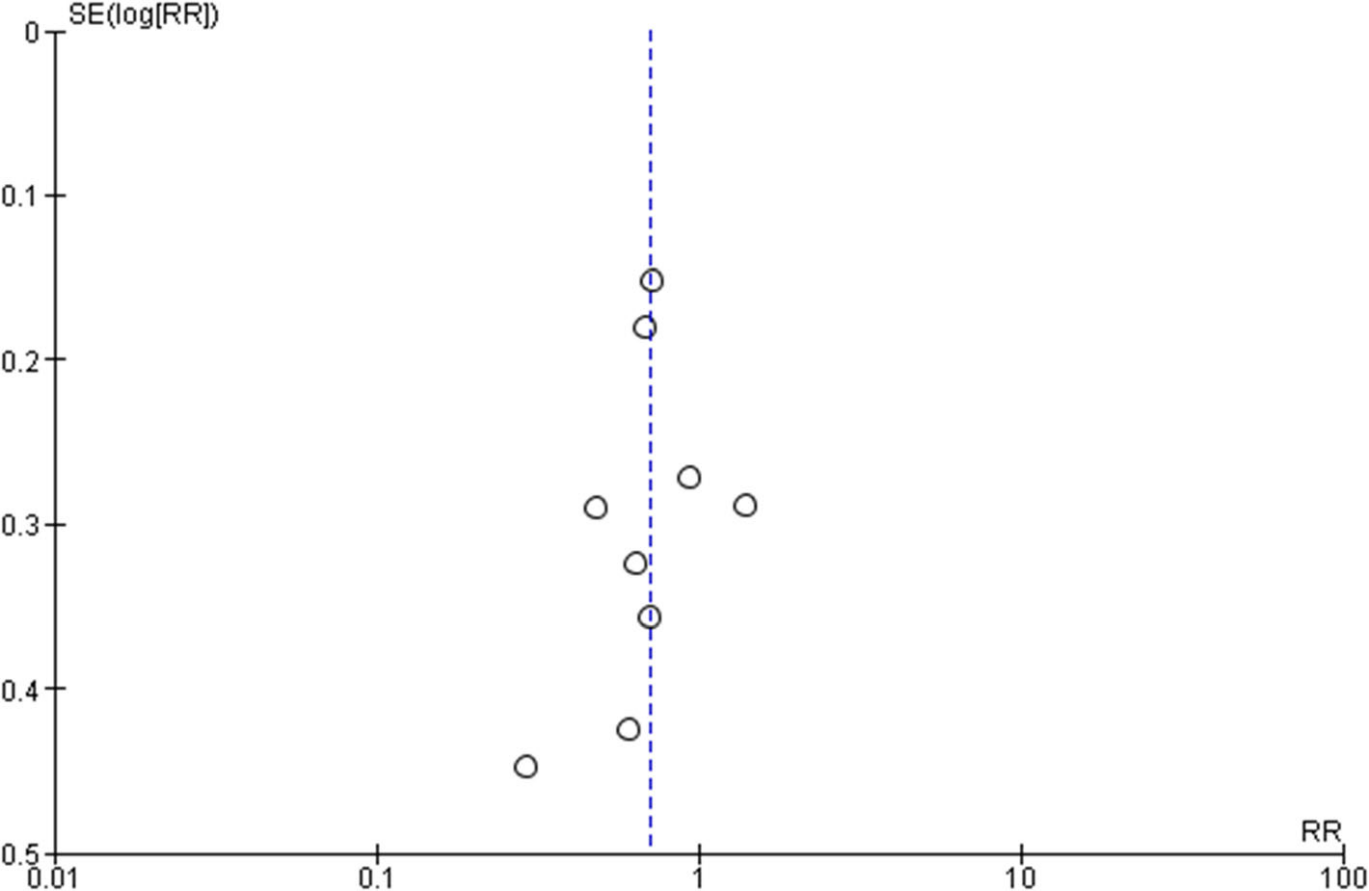
Funnel plot of incidence of VAP

A funnel plot was drawn for the primary outcome that is the incidence of VAP to determine the presence of possible publication bias. As can be seen in Fig. 14, there was no apparent publication bias as the funnel plot is symmetrical.

## Discussion

The current meta-analysis was planned to determine the effect of probiotics in the prevention of VAP by including randomized control trials on adults as patient populations. A total of nine studies were included in the meta-analysis and most of these studies had a low risk of selection, performance, reporting, and attrition bias. Few of the studies had a high detection bias risk. The forest plot analysis of the outcomes showed that probiotics had a good effect in reducing the incidence of VAP (*P* = 0.002), the duration of mechanical ventilation (*P* = 0.02), length of ICU stay (*P* = 0.001), and in-hospital mortality (*P* = 0.04). However, the use of probiotics did not affect the length of hospital stay (*P* = 0.47), the incidence of oropharyngeal colonization (*P* = 0.07), and the incidence of diarrhea (*P* = 0.06).

During the review, few assumptions were made as studies were inconsistent in reporting measures of association and differed in characteristics and timelines of endpoints. These assumptions were mostly related to the secondary outcomes of the review. We had used an empiric conversion for converting median reported values to mean using the equation. We did a sensitivity analysis excluding the studies for which this was done. We found that magnitude of effect size changes for the outcomes; duration of mechanical ventilation becomes non-significant from significant (−4.32 [−9.12, 0.49]) (*P* = 0.08) while the length of ICU stay, the mean difference still remained significant (−4.37 [−7.89, −0.85]) (*P* = 0.01). However, the direction of effect remained unaltered. The length of hospital stay remained non-significant −3.79 (−8.47.89; *P* = 0.11) on sensitivity analysis. Similar assumptions and its sensitivity analysis were not made in any of the previous meta-analysis.

We did subgroup analysis based on prior assumptions that effect estimates of probiotics may vary based on the quality of trials, population characteristics, and reporting of microorganism. However, we did not find evidence of interaction between postulated subgroups, and differences between the subgroups were non-significant implying the overall effect size estimates were consistent between subgroups both quantitatively and qualitatively. Bo et al. [25] in their meta-analysis also showed that even after the removal of studies with a high risk of bias probiotics still had a positive effect on the incidence of VAP.

A recent meta-analysis by Su et al. [26] published in 2020, showed that probiotic administration was associated with a statistically significant reduction in the incidence of VAP and a reduction in the duration of antibiotic use for VAP. However, two studies included in the meta-analysis [10, 11] did not compare the use of probiotics with placebo. The study by Oudhuis et al. [10] compared antibiotic use with probiotics in the reduction of VAP rate while the study by Klarin et al. [11] compared the use of probiotics with chlorhexidine mouth wash. Also, two studies included in the meta-analysis-Spindler Vessel et al. [12] and Forestier et al. [13] reported pneumonia which may not be ventilator associated. Thus, the above meta-analysis may not be determining the effect of probiotics on VAP accurately. For our meta-analysis, the protocol and trial of one large multicenter study by Deborah Cook et al. [27] were found eligible. Since the results were not published, the authors were mailed to share the results, but the same was not shared.

Our meta-analysis shows that the administration of probiotics significantly decreases the incidence of VAP, the duration of mechanical ventilation, length of ICU stay, and in-hospital mortality compared to placebo. The decrease in the incidence of VAP after probiotic administration is consistent with the previous meta-analysis by Su et al. [26], Weng et al. [14], Chen et al. [5], Liu et al. [28], Manzanares et al. [29], Siempos et al. [30], Bo et al. [8], and Banupriya et al. [31]. However, two meta-analyses, Gu et al. [32] and Wang et al. [33] did not show a statistically significant decrease in the incidence of VAP after probiotic administration.

In our study, no statistically significant decrease was seen in the length of hospital stay, the incidence of diarrhea, and the incidence of oropharyngeal colonization. However, a statistically significant reduction in the duration of mechanical ventilation, length of ICU stay, and in-hospital mortality was seen in our study which was not reported in other meta-analyses [5, 14, 25, 26, 28–33]. A meta-analysis conducted by Siempos et al. in 2010 [30] showed a reduction in length of ICU stay and respiratory tract colonization by *Pseudomonas aeruginosa*. Gu et al. in 2014 [34] also showed a reduction in length of ICU stay with the administration of probiotics. However, the meta-analysis by Siempos et al. [30] is old and new RCTs have been reported after that. The meta-analysis by Gu et al. in 2014 [34], described the length of ICU stay in only two studies which is a statistically insignificant number.

The absence of any effect on the other secondary outcomes in our meta-analysis could be due to the variability in the populations studied, the probiotic agents used, doses, time points when therapy was initiated, durations of therapy, the routes of administration, and the diagnostic criteria used for establishing VAP.

The definition of VAP used in the included RCTs was variable. In two of the RCTs [7, 16], two VAP rates were given, microbiological as well as clinical VAP. Of the two, the microbiological definition of VAP was used for our meta-analysis, as this is the definition used most consistently by many authors in various RCTs/meta-analysis. Thus, VAP definition is an important limitation of our meta-analysis as we relied on the reported definitions; a uniform definition is lacking in the RCTs. Large multicentric RCT with a uniform objective definition of ventilator-associated event (VAE) needs to be done in the future to precisely evaluate the effect of probiotics on VAP. VAE, as defined by CDC, is said to happen if after a period of stability or improvement, the patient has worsening oxygenation (minimum FiO_2_ increases by ≥ 0.2 or minimum daily PEEP increases by ≥ 3 cm H_2_O) [35].

To calculate for the incidence of oropharyngeal colonization, the rate of colonization at day 7 of ICU stay was selected. The definition of diarrhea as defined in most studies was ≥ 3 liquid stools/day [7, 19, 22].

There are few limitations of the meta-analysis. Firstly, the type, duration, and mode of administration of probiotics in the various RCTs were not constant among the various RCTs. The treatment duration in few studies was too short for any concrete evidence. Secondly, the diagnosis of ventilator-associated pneumonia was based on varied definitions in the RCTs (as listed in the table) with the element of subjectivity. Though the recent CDC definition of VAE is more objective, but, it is not yet used by any of the published RCTs on probiotics.

Thirdly, we were unable to assess the impact of probiotics on other clinically important endpoints: length of antibiotic therapy and antibiotic consumption. This is because of sparse and inconsistent reporting of the above endpoints across trials. Fourth, RCTs included in the meta-analysis have excluded immunocompromised patients. Thus, the role of probiotics in this important patient population cannot be ascertained. Furthermore, no study reported any side effects of probiotics use.

Thus, a large, multicentric, randomized control trial evaluating the use of probiotics (optimal type, dose, and route of administration) for VAP in an immunocompromised patient population is needed which should also evaluate the possible side effects of probiotics. The trials can also evaluate the changes in the microbiome following critical illness and the effect of probiotics/synbiotics on restoring a healthy microbiome in treated patients.

The strength of this current systematic review includes the use of standard methods to reduce bias (comprehensive literature search, duplicate data abstraction, specific criteria for searching and analysis), and the analysis of relevant clinical outcomes in the critically ill. Additional conduct of explicit subgroup and sensitivity analysis provides evidence in the robustness of estimates.

## Conclusion

It can be concluded that the use of probiotics reduces the incidence of VAP, duration of mechanical ventilation, length of ICU stay, and in-hospital mortality but has no effect on the length of hospital stay, incidence of diarrhea, and incidence of oropharyngeal colonization. The benefit of probiotics seems clinically plausible, as the effect estimates were favoring probiotics in most above clinically related endpoints. However, the varying definitions and subjectivity of VAP criteria preclude true estimates of effect. An objective uniform definition of VAP and large scale and large multicentric randomized controlled trials are needed to evaluate the further optimal type, dose, and route of administration for probiotics in ventilator association pneumonia.

## Data Availability

The datasets used and/or analyzed during the current study are available from the corresponding author on reasonable request.

## Abbreviations

VAP: Ventilator-associated pneumonia
ICU: Intensive care unit
USD: Unites States dollar
SDD: Selective digestive decontamination
SOD: Selective oral decontamination
RCT: Randomized controlled trial
PB: Priyam Batra
KDS: Kapil Dev Soni
PM: Purva Mathur
SD: Standard deviation
IQR: Interquartile range
CDC: Center for disease control and prevention
